# Human saliva as a source of anti-malarial antibodies to examine population exposure to *Plasmodium falciparum*

**DOI:** 10.1186/1475-2875-10-104

**Published:** 2011-04-29

**Authors:** Patricia Tabernero Estévez, Judith Satoguina, Davis C Nwakanma, Sheila West, David J Conway, Chris J Drakeley

**Affiliations:** 1 Faculty of Infectious & Tropical Diseases, London School of Hygiene and Tropical Medicine, London, UK; 2 Medical Research Council laboratories (UK), Fajara, The Gambia; 3 University of Abomey-Calavi, LBBM, Cotonou, Benin; 4 Wilmer Eye Institute, John Hopkins School of Medicine, Baltimore, USA

## Abstract

**Background:**

Antibody responses to malaria antigens reflect exposure to parasites, and seroprevalence correlates with malaria transmission intensity. Antibodies are routinely measured in sera or on dried blood spots but a non-invasive method would provide extra utility in sampling general populations. Saliva is already in use in the detection of plasma-derived IgM and IgG to viral infections. In this study, antibodies to *Plasmodium falciparum *merozoite antigens were compared between blood and saliva samples from the same individuals in unlinked surveys conducted in Tanzania and The Gambia.

**Methods:**

In Tanzania, 53 individuals provided paired fingerprick blood and saliva sample using two commercially available sampling devices. In the Gambia, archived plasma and saliva samples collected from 200 children in the Farafenni area in a cross-sectional survey were analyzed.

IgG antibodies against *P. falciparum *antigens, Merozoite Surface Protein-1 (MSP-1_19_) and Apical membrane Antigen (AMA-1) were measured by ELISA in paired saliva and blood samples from both sites. Antibody levels were compared as continuous optical density (OD) values and by sero-positivity.

**Results:**

Significant correlations between saliva and plasma antibody levels were seen in Tanzania for both antigens, AMA-1(r^2 ^range 0.93 to 0.89, p < 0.001) and MSP-1_19 _(r^2 ^range 0.93 to 0.75, p < 0.001), with a weaker correlation for results from The Gambia (r^2^range 0.64 to 0.63, p < 0.01). When assessed as seropositivity and compared with plasma, sensitivity and specificity were good with saliva antibody levels to both AMA-1 and MSP-1_19 _(sensitivity range 64-77% and specificity range 91-100% & 47-67% and 90-97% respectively) over the different sample sets.

**Conclusions:**

These data demonstrate anti-malarial antibodies can be detected in saliva and correlate strongly with levels in plasma. This non-invasive relatively simple collection method will be potentially useful for general population surveys, and particularly in migratory populations or those with infrequent contact with health services or opposed to blood withdrawal. Further studies will be needed to optimize collection methods, standardize volumes and content and develop controls.

## Background

The strengthening of control efforts has successfully reduced malaria burden in many countries and reawakened discussions of malaria elimination[1]. Whilst conjecture remains about whether elimination can be achieved there is a need to more accurately define malaria exposure at the low levels of transmission, which will inevitably be encountered if control measures succeed[2-4]. More accurate estimates of exposure and transmission intensity will allow the evaluation of the impact of control activities and deployment of future control methods[5].

Parasite rate (PR) and the entomological inoculation rate (EIR) are the measures widely used to estimate the transmission intensity for malaria, but these have poor precision at low transmission levels[6]. It has previously been shown that anti-malarial antibodies as age specific seroconversion rates are an effective tool to assess malaria endemicity and burden of the disease[5,7-9]. Antibodies can persist for months or years after infection and, therefore, may have particular utility as a proxy measure of malaria transmission in low transmission settings [6].

Samples for both PR and serological estimations are typically collected as blood by finger prick. However, drawing blood involves risk of accidental infections (albeit minor) in resource-poor environments and presents challenges in communities with blood taboos [10,11]. One alternative is oral fluid which is a mixture of IgA-rich saliva and IgG-rich crevicular fluid (a transudate of serum expressed at the crevice between teeth and gums) and is already a substitute of serum samples in the diagnosis of several pathogens, such as HIV[12,13]. Commercial saliva-based kits for HIV and illicit drugs are already available for the detection of human antibodies for population-based studies[12,14].

Plasmodium DNA has been successfully detected in saliva samples [15-17] and the additional detection of antibodies would be a further considerable benefit in developing rapid, safe and affordable approach to determine exposure to and infection with *Plasmodium falciparum*. The objective of this study was to evaluate oral fluid as an alternative to blood collection for the detection of anti-malarial antibodies. The study reports results from a prospectively designed collection of paired plasma and saliva samples in Tanzania and retrospective analysis with the same ELISA methodology of archived plasma and saliva samples from a previously conducted study in The Gambia[15].

## Methods

### Study sites and conduct: Tanzania

The study was conducted in July 2009 in rural-central Tanzania in the villages of Ihanda and Ndurugumi, in Dodoma Region. In this area, malaria is hypoendemic with transmission occurring primarily during and immediately after the rainy season, from January to March. This survey was conducted over a two week period July 2009 and nested within a larger study investigating the impact of azithromycin for Trachoma treatment on malariometric indices. The eligible subjects for the study were parents with a child under five years old and these were included on a first come first serve basis during the survey period. The aim was to compare antibody responses to malaria antigens in saliva collected with two commercially available saliva collection devices with antibody responses in plasma from the same individuals. None of the participants had clinical symptoms of malaria at the time of the survey. This project received ethical approval from the Ethical Committees of the National Institute for Medical Research in Tanzania and the London School of Hygiene and Tropical Medicine. Individual informed consent was obtained in Swahili from the enrolled individuals.

### Study site and conduct: The Gambia

In The Gambia, samples were collected in a cross sectional malaria survey in August 2008. The primary aim of the study was to evaluate the sensitivity of parasite DNA detection in saliva for diagnosing malaria. Salivary anti-malarial antibodies were measured in a second analysis. Following informed consent from either the parent or guardian of eligible study participants, a baseline malaria screening of children aged 1 to 15 years was conducted in seven villages around the Farafenni area. In the Gambia malaria is seasonal, occurring mainly during the rainy season from July to November with a peak in September. The study was jointly reviewed and approved by the Gambian Government-Medical Research Council Laboratories Ethics Committee and the Programme for Appropriate Technology in Health (PATH) Research Ethics Committee (REC) USA.

### Sample collection and processing: Tanzania

On completion and signing of the informed consent, saliva was taken from the parent and then the parent obtained the sample from the child. Oral fluid was collected from each participant using two different commercially available devices, Oracol (Malvern Medical Developments Limited, Worcester, U.K.) and OraSure-Intercept (OraSure Technologies, Inc. Bethlehem, PA, US). These devices have previously been used in the surveillance of viral and bacterial diseases [10,18-20] and were selected in order to better understand the efficiency of anti-malarial antibody elution from human saliva. Oracol was used as previously described[19,21,22]. Timers were used to be consistent with the time when collecting the saliva samples in each individual with a maximum of two minutes per swab allowed. Samples were initially stored in a cool box prior to longer-term storage at -20 degrees. For both devices, the specimens were centrifuged at 1000 g/min for 5 min[18,19,21], swabs were discarded and the extracted saliva was pipetted into a 1.5 ml tube and stored at +4°C until testing.

The blood samples were collected by finger prick on to Whatman 3 M filter paper, dried with silica gel and stored at +4°C until testing. The reconstitution of dried blood spots was conducted as previously described [8]. Briefly, a 3.5 mm diameter circle was punched out from a blood spot and reconstituted by adding 300 μl of the reconstitution buffer made from PBS/Tween wash solution and 0.1% Azide after overnight agitation.

### Sample collection and processing: The Gambia

Approximately 1 ml of saliva was collected directly into sterile 50 ml falcon tube by spitting and concurrently, 250 μl of finger prick blood was collected into EDTA microtainer tube from each study participant. Samples were temporarily kept in a cold box in the field and processed for storage at the laboratory within 2H of collection. The plasma fraction was separated from whole blood by centrifugation at 2,500 rpm while the saliva samples did not undergo further processing. All samples were stored at -20°C until serological assays were carried out.

### Serological assays

A quantitative enzyme-linked immunosorbent assay (ELISA) was used to analyse oral fluid and blood samples to detect IgG antibodies to recombinant blood stage *P. falciparum *malaria antigens AMA-1(3D7) and MSP1_19 _(Wellcome) [23] and conducted as previously described [6,8]. Briefly, Immulon-4 plates (Nunc) were coated overnight with 50 μl of 0.5 μg/ml the antigen. After blocking 50 μl of each of the samples and standards (a pool of positive sera from The Gambia) were added to duplicate wells; filter paper eluate and plasma were tested at a final dilution of 1:1,000 for MSP-1_19 _and 1:2,000 for AMA-1. Saliva was used undiluted. The plates were incubated over night at 4C after which rabbit anti-human IgG-antibody conjugated to horse-radish peroxidase (Dako) was added at a dilution of 1:5,000. Antibody responses were detected as optical densities (OD) after development with o-phenylenediamine (OPD) and reading at a wavelength of 490 nm on a spectrophotometer. In the absence of known positive and negative saliva samples serial dilutions of a pool of positive sera control was included on each ELISA plate together with a non-immune control pool as negative control.

### Statistical analysis

ELISA OD were converted to antibody titres expressed in Arbitrary Units (AU/ml) using the standard curve from a pool of hyperendemic sera. Paired t-tests of the continuous data were conducted and correlation coefficients calculated. A mixture model was used to define an arbitrary cut-off for positivity for each sample collection method [8]. Briefly, the distribution of normalized OD values was fitted as the sum of two Gaussian distributions (a narrow distribution of sero-negatives and a broader distribution of sero-positives) using maximum likelihood methods. The mean OD of the Gaussian corresponding to the sero-negative population plus three standard deviations was used as the cut-off for sero-positivity. A separate cut off was generated for each antigen and each sample type (plasma & saliva). Sensitivity analysis was conducted in Stata11 (Statacorp, Texas).

## Results

**Figure 1:**
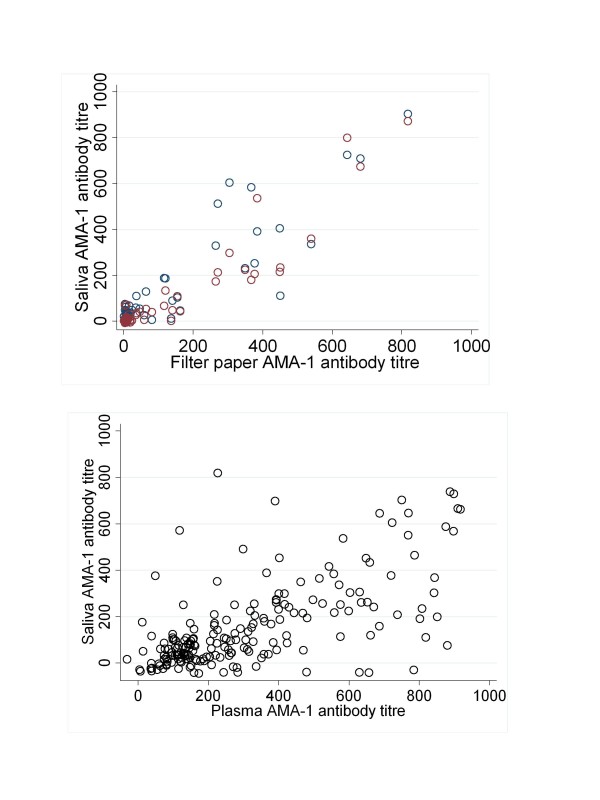
**Scatterplots showing relationship between paired antibody titres from plasma and saliva to *P. falciparum *merozoite surface antigen AMA-1 in a)Tanzania and b) The Gambia**. In plot a) open red circles represent measures by OraSure and blue circles by OraCol.

Matched filter paper or plasma samples and saliva were available from 53 Tanzanian participants and 200 Gambian participants. Antibody titre results from saliva compared well with those from plasma in both settings and for all antigens. In Tanzania AMA-1 titres showed highly significant correlation between the two fluids and with both oral fluid devices (r²Oracol-fingerprick = 0.89, r²Orasure-Fingerprick = 0.93, both p < 0.001, figure 1a &1b, Table 1). Similar results were seen for antibodies against MSP-1_19 _(r² Oracol-fingerprick = 0.75, r²Orasure-Fingerprick = 0.94, both p < 0.001, figure 1c &1d, Table 1). Data from The Gambia also showed significant correlations for all antigens tested though overall correlation coefficients were lower than those observed in Tanzania (r²AMA-1 = 0.64, r²MSP-1_19 _= 0.63 both p < 0.01). Analysis of antibody titres found that mean titres were significantly different between plasma and saliva for all antigens and in both settings except for the saliva collect via the Oracol sampling device and fingerprick plasma (Table 1.)

**Table 1:** Comparison of antibody titres and prevalence measured in plasma and saliva against MSP-1_19 _and AMA-1 *P. falciparum *merozoite surface antigens

	*Tanzania (n = 53)*			*The Gambia (n = 200)*	
**Antigen**	**Plasma**	**Saliva** **Oracol**	**Saliva** **Orasure**	**Plasma**	**Saliva**

AMA-1 R^2^		0.89	0.93		0.64
Mean response (range)	138 (1-817)	148 (3-902)	108* (0-871)	317 (0-917)	185* (45-818)
% positive	47	34	36	36	30
MSP-1_19_ r^2^		0.75	0.93		0.63
Mean response (range)	61 (0-769)	62 (0-625)	37* (0-610)	229 (0-844)	113* (0-612)
% positive	28	26	15	30	20

* = significant difference between means of titre response in saliva and plasma using t-test

ND not done

**Figure 2:**
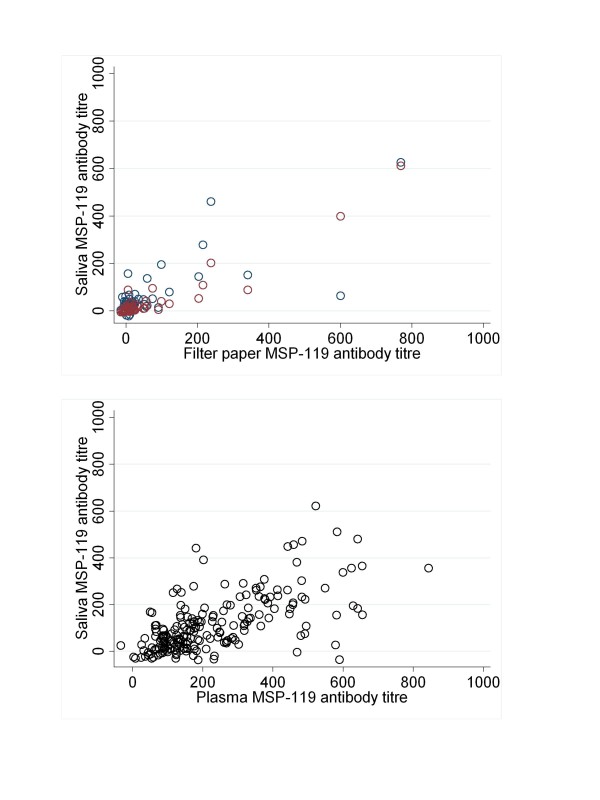
**Scatterplots showing relationship between paired antibody titres from plasma and saliva to *P. falciparum *merozoite surface antigen MSP-1_19 _in a) Tanzania and b) The Gambia**. In plot a) open red circles represent measures by OraSure and blue circles by OraCol.

When antibody levels were classified as positive or negative, prevalences detectable from plasma were significantly higher in all but one comparison (Tanzania MSP1-_19 _between filter paper and the saliva collected using Orasure). Using the fingerprick derived plasma antibody prevalence as the gold standard, sensitivity of the saliva test is presented in Table 2. Specificity was uniformly high (> 90%), but sensitivity varied greatly (46-76%) though the ROC (receiver operating characteristic curve; the average of sensitivity and specificity) was consistently 0.72 or above.

**Table 2:** Sensitivity, specificity & ROC* for saliva derived seroprevalence to *P. falciparum *merozoite surface antigens

	Sensitivity (95% CI)	Specificity (95% CI)	ROC (95% CI)
AMA-1			
Tanzania Oracol	76.7 (54.9-90.6)	100 (87.7-100)	0.88 (0.8-0.96)
Tanzania Orasure	64 (42.5-82)	92.9 (76.5-99.1)	0.78 (0.68-0.89)
The Gambia	68 (56-78.8)	91.2(85-95.6)	0.80(0.74-0.86)
MSP-1_19_			
Oracol	46.7 (21.3-73.4)	97.4 (86.2-99.9)	0.72 (0.58-0.85)
Orasure	66.7 (38.4-88.2)	90.0(75.2-97.1)	0.78 (0.64-0.91)
The Gambia	53 (40.1-66.5)	94.2(89-87.5)	0.74(0.67-0.81)

* ROC = receiver operating characteristic curve; the average of sensitivity and specificity

Fingerprick derived plasma antibody prevalence was the gold standard for sensitivity and specificity analysis.

## Discussion

This study aimed to determine whether anti-malarial antibodies could be detected in saliva by indirect ELISA and if the detected antibodies correlated with those from plasma either collected directly or on filter papers. Results indicate good correlations between saliva and serum antibody titres for both antigens tested and weaker correlations between sero-prevalence. Given that these data are derived from two studies in different countries whose only consistency of protocol was the ELISA assay for antibody detection, the findings suggest that this innovative and non-invasive sampling method has some potential for population-based serological surveys for malaria.

Saliva, rich in serum-derived IgM and IgG, represents a convenient alternative sample collection method to serum, particularly useful when repeated blood sampling may not be possible [10]. Saliva collection has several advantages: it does not involve the usage of sharps, it is easy to collect and store and could be used to access communities with blood taboos [24]. It is also likely to be more ethically acceptable for repeat sampling of the same individual. From a logistical standpoint, the methods used in this study could be easily transferred to other laboratories where epidemiological studies are conducted[20].

However, there are several potential limitations that need further investigation. Whilst correlations were good, the overall titres were significantly lower in saliva, as expected. This difference was most pronounced in assays conducted in The Gambia and are perhaps a result of a combination of using directly collected plasma and a non standardized saliva collection method (though collection of saliva via spitting is a simple approach). Plasma levels of hormones and antibodies are often routinely higher than those in saliva and work is required to evaluate optimal comparative dilutions [12]. The sensitivity and thus interpretation of a saliva based ELISA for detection of trypanosomiasias antibodies was found to be highly dependent on saliva dilution with a 1:20 dilution providing acceptable result and those of 1:40 not [25]. Clearly work is needed to standardise the amount and 'quality' of saliva collected. Even in Tanzania when two similar specific saliva collection methods were used, Oracol and Orasure, differences in titres were observed. These probably reflect the way each of the swabs were used as well as the storage buffer and the material they are made of and the relative recovery of saliva from the swabs [19,26]. Although both saliva devices yielded saliva samples suitable for the qualitative determination of malaria specific IgG antibody, these differences would require standardization for wider use. There is also significant variation in the amount of saliva produced by individuals [10]. This is compounded by the further dilution of samples with buffer a necessary addition to stabilize and preserve saliva during field collection. The identification of a standard protein against which saliva volume could be calculated would be useful. Human serum albumin is one option used in serum and alternatives for saliva need to be investigated. Similarly, defining a positive control saliva for use in future assays will be necessary.

The relationship between plasma and saliva antibody titre and prevalence differed slightly depending on the target antigen though this relates to the higher immunogenicity of AMA-1 and the antibody titre it induces. The greatest discordance between plasma and saliva titres was observed with the lowest values and this has been shown when comparing anti-malarial antibodies derived from different sources [8]. Importantly, for the antigens used in this study which have been most widely utilized in recent times as markers of exposure, [27-29], correlations were good. Indeed, combining seropositivity to both antigens in this data set increases overall sensitivity, specificity and ROC to some degree (for the Oracol device in Tanzania 70.4, 96.2 and 0.83, respectively and for The Gambia 67.2, 92.5 and 0.8, respectively). These ROC values reflect only fair accuracy but should improve with optimization and using these antigens together, ideally in a single ELISA to minimize workload, would likely provide a better measure of exposure. This study was not designed to compare results between the two study sites. However, difference in the survey methodologies used, including the different age range of the individuals recruited, will account for the some of the variability of the correlation results observed between the two sites.

This study opens the way for more extensive studies to assess the potential of human saliva in the detection of anti-malarial antibodies. Seroprevalence data from saliva has already been used to describe the immuno-epidemiology of populations for meningococcal infection, tetanus and other diseases for many years [10,11,20,22,30,31]. Moreover, malaria parasite DNA can also be detected from saliva [15] allowing an assessment of both exposure and infection from a saliva sample. Further research in a longitudinal study would help to determine if anti-malarial saliva antibodies are influenced by food, tobacco or other diseases and could be extended to study exposure to other malaria species, such as *Plasmodium vivax*.

## Conclusions

Ultimately, a variety of different diagnostic approaches are needed to deal with the different epidemiological and sociological scenarios in malaria. These preliminary data suggest saliva represents a potential alternative to blood for the assessment of anti-malarial antibody levels in population surveys. Further studies confirming and standardising the recovery of antibodies are required.

## Competing interests

The authors declare that they have no competing interests.

## Authors' contributions

PTE conducted the field work in Tanzania with SW, analysed the samples and wrote the first draft of the report. DCN, DJC and JS designed the study in the Gambia and JS conducted the ELISA. CD conceived the project and wrote the paper with contributions from all authors. All the authors read and approved the final manuscript.
